# Multimodal ultrasonographic assessment of leiomyosarcoma of the femoral vein in a patient misdiagnosed as having deep vein thrombosis: A case report

**DOI:** 10.1097/MD.0000000000008581

**Published:** 2017-11-17

**Authors:** Mei Zhang, Feng Yan, Bin Huang, Zhoupeng Wu, Xiaorong Wen

**Affiliations:** a Department of Ultrasound; b Clinical Ultrasound Imaging Drug Research Lab; c Department of Vascular Surgery, West China Hospital, Sichuan University, China.

**Keywords:** common femoral vein, leiomyosarcoma, multimodal ultrasonography, thrombus

## Abstract

**Rationale:**

Primary leiomyosarcoma (LMS) of the vein is a rare tumor that arises from the smooth muscle cells of the vessel wall and has an extremely poor prognosis. This tumor can occur in vessels such as the inferior vena cava, great saphenous vein, femoral vein, iliac vein, popliteal vein, and renal vein; the inferior vena cava is the most common site. LMS of the femoral vein can result in edema and pain in the lower extremity; therefore, it is not easy to be differentiated from deep vein thrombosis (DVT). Moreover, virtually no studies have described the ultrasonographic features of LMS of the vein in detail.

**Patient concerns:**

We present a case of a 55-year-old woman with LMS of the left femoral vein that was misdiagnosed as having deep vein thrombosis (DVT) on initial ultrasonographic examination. The patient began to experience edema and pain in her left leg seven months previously. She was diagnosed as having DVT on initial ultrasonographic examination, but the DVT treatment that she had received for 7 months failed to improve the status of her left lower limb.

**Diagnoses:**

She subsequently underwent re-examination by means of a multimodal ultrasonographic imaging approach (regular B-mode imaging, color Doppler imaging, pulsed-wave Doppler imaging, contrast-enhanced ultrasonography), which confirmed a diagnosis of LMS.

**Interventions:**

This patient was treated successfully with surgery.

**Outcomes:**

This case demonstrates that use of multiple ultrasonographic imaging techniques can be helpful to diagnose LMS accurately. Detection of vasculature in a dilated vein filled with a heterogeneous hypoechoic substance on ultrasonography is a sign of a tumor.

**Lessons:**

The pitfall of misdiagnosing this tumor as DVT is a useful reminder.

## 1 Introduction

The majority of leiomyosarcomas (LMS) originate in the muscle layers of the gastrointestinal tract or uterus. Primary LMS of the vein is a rare tumor that arises from the smooth muscle cells of the vessel wall. Al-Saif et al^[1]^ reported that LMS of the vein has an extremely poor prognosis, with 5-year malignancy-free survival of 30% to 50% after wide surgical resection. This tumor can occur in vessels such as the inferior vena cava, great saphenous vein, femoral vein, iliac vein, popliteal vein, and renal vein; the inferior vena cava is the most common site, according to the literature.^[2–8]^ LMS of the femoral vein can result in edema and pain in the lower extremity, and consequently, is not easy to be differentiated from deep vein thrombosis (DVT). Moreover, the ultrasonographic features of LMS of vein has been reported in some cases,^[9]^ but virtually no studies have described the ultrasonographic features of LMS of the vein in detail.

In this case, we used a multimodal ultrasonographic imaging approach (regular B-mode imaging, color Doppler imaging, pulsed-wave Doppler imaging, contrast-enhanced ultrasonography [CEUS]) and found LMS of the femoral vein, which had been misdiagnosed as DVT. The pitfall of misdiagnosing this tumor as DVT is a useful reminder.

## 2 Consent

The patient signed the necessary documents to consent to the use of her data for teaching and publication.

## 3 Case report

### 3.1 Case history

A 55-year-old woman was admitted to the Vascular Surgery Department of our hospital for ultrasonographic examination of suspected LMS in the left common femoral vein. Seven months previously, the patient had begun to experience edema and pain in the left leg, and underwent a rapid ultrasonographic evaluation in the emergency department of our hospital. Based on the ultrasonography, thrombus in the left common femoral vein and superficial femoral vein was diagnosed. The patient was administered anticoagulation therapy, with no improvement of the status of the lower limb. Before anticoagulation therapy, coagulation factors, fibrinogen level, and C-reactive protein (CRP) level were normal. During anticoagulation therapy, prothrombin time was 20.4 s (reference value, 9.6–12.8 s) and International Normalized Ratio was 1.8 s (reference value, 0.86–1.14 s), while other laboratory data, including fibrinogen level, thrombin time, activated partial thrombin time, and CRP level, were normal. She underwent a repeat ultrasonographic examination after 7 months, which confirmed that a tumor, rather than a blood clot, had involved the left external iliac vein, common femoral vein, deep femoral vein, and superficial femoral vein. Physical examination revealed a mass with a diameter of approximately 2 cm in the left inguinal region, which could not be pushed, mild cyanosis, and swelling of the left leg.

### 3.2 Ultrasonographic findings

The first ultrasonographic examination (Philips IU22; Bothell, WA; probe L9-3, 3–9 MHz; venous condition) performed 7 months previously in the emergency department had revealed dilation of the common femoral vein (diameter, 18 mm), as well as a hypoechoic mass in the lumens of the left external iliac vein, common femoral vein, deep femoral vein, and superficial femoral vein. Color Doppler flow imaging revealed no color flow signal in the lumen of the common femoral vein. Thus, the patient was diagnosed as having DVT.

**Figure 1:**
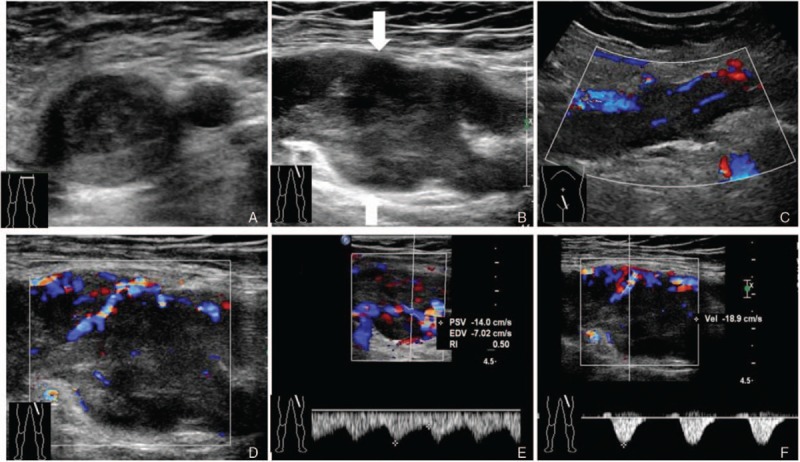
Gray-scale and color Doppler ultrasonographic findings of leiomyosarcoma. High-resolution gray-scale images of a cross-section (A) and longitudinal section (B) show the dilation of the common femoral vein (diameter, 25 mm) and a heterogeneous hypoechoic mass in the lumen (A–D). Color Doppler flow imaging shows the rich blood flow in the hypoechoic mass in the left external iliac vein (C) and common femoral vein (D). Pulsed-wave Doppler imaging reveals the low- and high-resistance arterial flow, PSV: 14.0 cm/s, EDV: 7.02 cm/s, RI: 0.50 (E) and PSV: 18.9 cm/s, EDV: 0 cm/s, RI: 1.00 (F), respectively. EDV = end diastolic velocity, PSV = peak systolic velocity, RI = resistance index, Vel = velocity.

**Figure 2:**
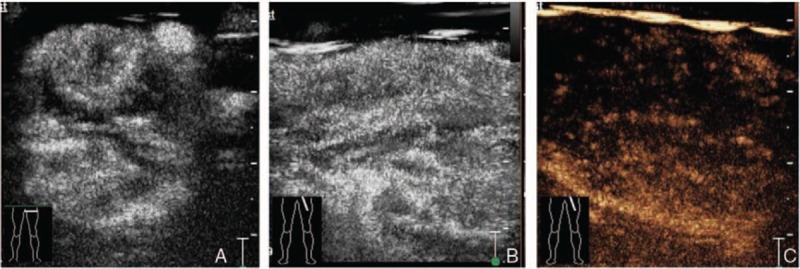
Contrast-enhanced ultrasonographic findings of leiomyosarcoma. Contrast-enhanced images of a cross-section (A) and longitudinal section (B) show the rapid, uneven, high levels of enhancement in the arterial phase. Contrast-enhanced imaging of a longitudinal section (C) reveals the low levels of enhancement in the venous phase.

In the second examination, we used a multimodal ultrasonographic imaging approach (Philips IU22; probes C5-1, 1–5 MHz; L9-3, 3–9 MHz; L12-5, 5–12 MHz; superficial condition). High-resolution gray-scale images further revealed a dilation of the common femoral vein (diameter, 25 mm), as well as a hypoechoic mass in the lumens of the left external iliac vein, common femoral vein, deep femoral vein, and superficial femoral vein (Fig. 1A and B). Color Doppler flow imaging showed rich color spots and linear blood flow signals in the hypoechoic mass (Fig. 1C and D). Pulsed-wave Doppler imaging detected low- and high-resistance arterial flow, peak systolic velocity (PSV): 14.0 cm/s, end diastolic velocity (EDV): 7.02 cm/s, resistance index (RI): 0.50 (Fig. 1E) and PSV: 18.9 cm/s, EDV: 0 cm/s, RI: 1.00 (Fig. 1F), respectively. CEUS revealed rapid, heterogeneous, high levels of enhancement in the arterial phase (Fig. 2A and B) and the low levels of enhancement in the venous phase (Fig. 2C), after injection of 2.4 mL of the contrast agent, SonoVue (Bracco, Milan, Italy). These findings confirmed that the initially diagnosed blood clot was actually a tumor in the vein.

### 3.3 Contrast-enhanced computed tomographic findings

**Figure 3:**
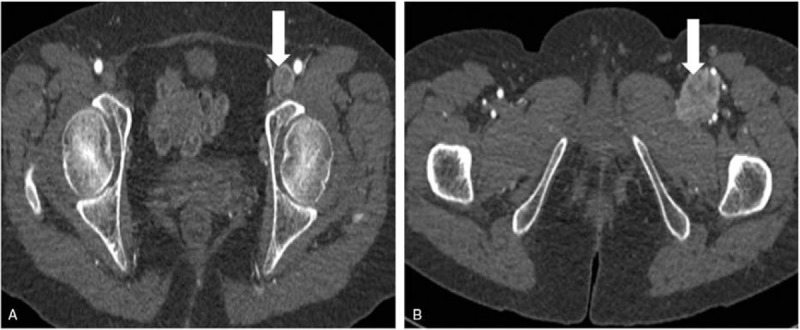
Contrast-enhanced computed tomographic findings of leiomyosarcoma. Contrast-enhanced imaging shows a partial venous enlargement due to the presence of a heterogeneous mass of soft tissue density in the lumen. The mass shows an obvious heterogeneous enhancement in the arterial phase. (A) Left external iliac vein and (B) common femoral vein.

After the second ultrasonographic examination, the patient underwent contrast-enhanced CT (CECT) for confirmation, which revealed a partial enlargement of the left external iliac vein and common femoral vein due to a heterogeneous mass of soft tissue density filling their lumens. The mass showed an obvious heterogeneous enhancement in the arterial and venous phases (Fig. 3A and B).

### 3.4 Surgical findings

**Figure 4:**
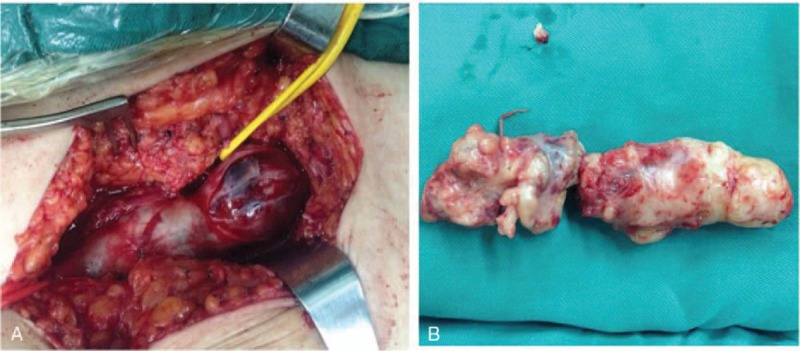
Surgical findings of leiomyosarcoma. (A) An obvious dilation extending from the left external iliac vein to the common femoral vein bifurcation. (B) A fish flesh-like, solid substance in the venous lumen.

**Figure 5:**
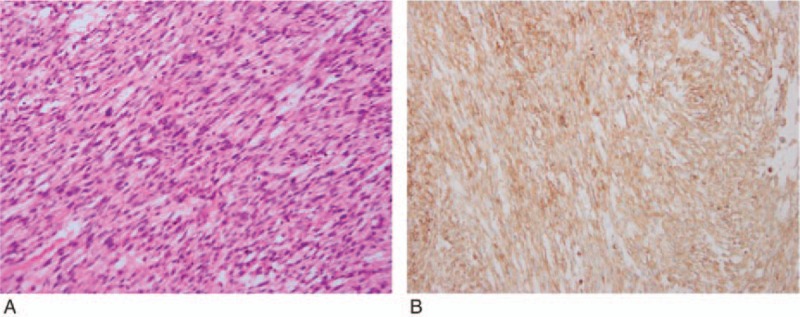
Pathologic findings of leiomyosarcoma. (A) Histopathologic examination reveals the spindle-shaped cells with the obvious nuclear pleomorphism and the frequent mitoses arranged in a beam pattern and partly in typical fascicles (hematoxylin and eosin stain, magnification ×200). (B) Immunohistochemical examination reveals the spindle-shaped cells showing immunopositivity for smooth muscle actin (magnification ×200).

During surgery, we found a mass extending from the left external iliac vein to the common femoral vein bifurcation, causing an obvious enlargement of the lumens of the involved veins (Fig. 4A). When the above veins were opened longitudinally, a fish flesh-like, solid substance was found in the lumens (Fig. 4B). The tumor originated from the deep femoral vein and involved the left external iliac vein, common femoral vein, deep femoral vein, and the upper section of the superficial femoral vein. The mass was not closely adhered to the venous wall of the left external iliac vein or common femoral vein nor to the upper section of the superficial femoral vein. Pathologic examination identified the mass as LMS rather than a blood clot (Fig. 5A and B). The tumor was removed successfully, and autologous saphenous vein replacement was performed.

## 4 Discussion

LMS of the vein usually causes DVT-like symptoms, and thus, can be misdiagnosed as having DVT. In China and many other countries, ultrasonography is the preferred method for diagnosis of DVT because it is a convenient, fast, noninvasive, cost-effective, and real-time imaging technique. However, sometimes, routine ultrasonographic examination of DVT may not be performed correctly when the venous lumen cannot be fully compressed despite firm compression with the transducer probe. One ultrasonographic feature of acute venous thrombosis is increased venous diameter due to a hypoechoic substance filling the lumen. In the present case, repeat ultrasonography 7 months later showed a significantly increased venous diameter due to the initially detected hypoechoic mass that had later increased in size. In chronic venous thrombosis, however, the venous lumen usually gets smaller with time. Thus, the above unusual finding led us to question our earlier diagnosis, and we took a multimodal ultrasonographic imaging approach to make a more precise diagnosis. According to Bousquet et al,^[10]^ LMS of the inferior vena cava are usually >10 cm long, hypoechoic, and heterogeneous; a cystic component also can be found, which is estimated to be related to tumor necrosis. However, no cystic component was found in our patient.

LMS are generally hypervascular.^[11]^ Newly formed arteries are a key finding to differentiate between vascular tumor and thrombus. Color Doppler flow imaging is a ready-to-use technique during basic ultrasonographic examination of a thrombus. In fact, correct setting of Doppler parameters can detect intratumoral color Doppler signals and prompt contrast-enhanced imaging techniques, such as CEUS and CECT for confirmation. Due to the incorrect setting of Doppler parameters (Philips IU22; probe L9-3, 3–9 MHz; venous condition), the first ultrasonographic examination in the emergency department failed to detect intratumoral color Doppler signals and led to misdiagnosis as DVT. To better display blood flow in the tumor, we used a higher-frequency probe for scanning (Philips IU22; probe L12-5, 5–12 MHz; superficial condition). Color Doppler flow imaging in the superficial condition revealed rich color spots and linear blood flow signals in the mass, which are consistent with the findings reported by Perisano et al.^[11]^ Pulsed-wave Doppler imaging revealed the low- and high-resistance arterial flow, PSV: 14.0 cm/s, EDV: 7.02 cm/s, RI: 0.50 (Fig. 1E) and PSV: 18.9 cm/s, EDV: 0 cm/s, RI: 1.00 (Fig. 1F), respectively, which seem inconsistent with other reports. For instance, Ceyhan et al^[12]^ reported that Doppler ultrasonography demonstrated low-resistance arterial flow in LMS. We suspect that the emergence of the low-resistance arterial flow may be related to an arteriovenous fistula. If there was no arteriovenous fistula in the tumor, only high-resistance arterial flow would be found; however, there was no vascular murmur detected in our case. This suggests that despite Doppler ultrasonography playing a major role in detecting tumor vasculature, it might not be sensitive enough to detect tumor microvasculature.

With the development of new ultrasonographic imaging modalities, preoperative diagnosis by ultrasonography is possible. In China, CEUS has been widely applied for imaging of multiple organs, such as the liver, kidney, breast, and thyroid, and is particularly useful for the detection of tumor vasculature and microvasculature. Sidhu et al^[13]^ reported that CEUS has several advantages, including the absence of radiation and harmful effects on the kidney and thyroid, and easy accessibility. Doppler ultrasonography can be used if information regarding blood flow is required. However, Greis^[14]^ reported that Doppler ultrasonography can detect blood flow in only relatively large vessels, and cannot evaluate the blood flow in microvessels or tissue perfusion; contrast agents can be used to overcome these limitations. In the present report, CEUS revealed rapid, heterogeneous, high levels of contrast enhancement in the arterial phase, and low levels of enhancement in the venous phase. Thus, CEUS can be used to assess both blood flow in microvessels and tissue perfusion, which is not possible with Doppler ultrasonography.

These ultrasonographic findings (e.g., heterogeneous, hypoechoic material, dilated vein) are not typical signs of LMS. According to Hübsch et al,^[15]^ similar findings sometimes occur in thrombi or other tumors. Typical findings of LMS in our case included arterial blood flow signals in a heterogeneous, hypoechoic mass on color Doppler flow imaging, and a heterogeneously enhanced mass on CEUS. Our CEUS experiences suggest that tumor vasculature can be used to distinguish between tumor and thrombus.

Both CEUS and CECT can make a correct diagnosis of LMS in a vein. Regular high-resolution B-mode ultrasonography and color Doppler flow imaging may be superior to CT in showing local details of peripheral blood vessels and revealing hemodynamic information, but tend to have difficulties in showing the deeper blood vessels, such as the external iliac vein and inferior vena cava. Since CEUS is based on B-mode ultrasonography, it is subject to its limitations. For example, the image quality of CEUS is influenced by obesity and intestinal gas. On the other hand, CT, especially CECT, can provide comprehensive information, such as location, size, and extension of the tumor, as well as the relationship of the tumor with the surrounding tissues and organs, without being interfered by obesity and intestinal gas. Therefore, CECT is better than CEUS for examination of tumors in the external iliac vein. In our case, CECT was used to confirm the findings of CEUS. Both CEUS and CECT revealed the venous enlargement due to a heterogeneous mass of soft tissue density filling the lumen, and the obvious heterogeneous enhancement probably due to tumor heterogeneous perfusion and hemorrhage or necrosis, which are consistent with the findings reported in the literature.^[12,16,17]^

In order to correctly diagnose cases like this in the clinical setting, ultrasound-guided tissue biopsy also can be used. However, ultrasound-guided biopsy is an invasive examination. Taking into account that abundant image inspection was conducted (including CECT and multimodal ultrasonography), we disregarded ultrasound-guided biopsy and performed surgical resection.

A brief description of the general clinical presentations and mutimodal ultrasonographic features of this case has been included in a recent paper.^[9]^ However, the detailed descriptions of multimodal ultrasonographic features, CT discovery, surgical findings, and pathological findings and any images of the multimodal ultrasonography, CT, surgery, and pathology have never been reported.

## 5 Conclusion

In summary, our multimodal ultrasonographic imaging approach enabled preoperative diagnosis of LMS. The key point in our case was careful observation by an alert sonographer when suspecting the presence of a tumor in the left external iliac vein and femoral vein. In contrast, in patients with suspected acute thrombosis, various ultrasonographic techniques should be used to carefully look for signs of tumor vasculature. The pitfall of misdiagnosing LMS as DVT is a useful reminder.

## Acknowledgment

The authors thank Editage (www.editage.com) for English language editing.
